# A new case of an Holarctic element in the Colombian Andes: first record of *Cordyla* Meigen (Diptera, Mycetophilidae) from the Neotropical region

**DOI:** 10.3897/zookeys.520.6142

**Published:** 2015-09-16

**Authors:** Olavi Kurina, Sarah Siqueira Oliveira

**Affiliations:** 1 Institute of Agricultural and Environmental Sciences, Estonian University of Life Sciences, Kreutzwaldi st 5-D054, 51014 Tartu, ESTONIA; 2 Universidade Federal de Goiás, Campus II. Instituto de Ciências Biológicas, Departamento de Ecologia, prédio ICB1. CP 131, CEP 74001-970, Goiânia – GO, BRAZIL

**Keywords:** Diptera, Mycetophilidae, fungus gnats, *Cordyla*, Neotropical region, taxonomy, distribution

## Abstract

Three new species of Mycetophilidae – *Cordyla monticola* **sp. n.**, *Cordyla pseudopusilla* **sp. n.** and *Cordyla reducta* **sp. n.** – are described from the Colombian Andes, representing the first described species of *Cordyla* Meigen from the Neotropical region. Colour photos of their habitus, wing and terminalia are provided. The morphological affinities of male terminalia are discussed in a worldwide context. The distributional pattern of the genus clearly indicates a case of northern elements reaching the north-western region of the Neotropics that corresponds to a secondary extension of a Holarctic clade to the south.

## Introduction

Members of the monophyletic genus *Cordyla* Meigen, 1803 are well distinguished among fungus gnats (Diptera: Mycetophilidae) because of their considerably small size, strongly humpbacked habitus, reduced number of flagellar segments and swollen antepenultimate segment of palpus. The latter character is unique within fungus gnats worldwide, enabling immediate recognition while working with collections on the genus level. Further identification of the species is based on several body-characters with emphasis on the number of flagellomeres, and colour and length of the swollen palpal segment. However, as usual, the most important set of the species level morphological characters is that of the male terminalia. According to their structure, the genus has been divided into three subgeneric groups as defined by Kurina (2001). Tuomikoski (1966) transferred *Cordyla* to the tribe Exechiini within which it has a rather isolated position (Rindal et al. 2009, Ševčík and Kjærandsen 2012).

Thirty-nine species are described in the limits of the genus so far, viz. twenty-four from the Palaearctic region, ten from the Nearctic region, three from the Oriental region and two from the Australasian region (Kurina and Oliveira 2013 and references therein). From undescribed species, the genus has also been known in the Neotropical region (Oliveira et al. 2007, Vockeroth 2009, Kurina and Oliveira 2013).

Over recent years the junior author has accumulated *Cordyla* specimens collected in Colombian Andes. The aim of this paper is to describe, illustrate and discuss three new *Cordyla* species from that material representing the first named species in the Neotropical region.

## Material and methods

All material was collected with Malaise traps from the Colombian Andes at an altitude greater than 1900 m a.s.l. from 2001 to 2003. The collecting was performed during “The Colombian Arthropod Project (CAP)” – a collaborative arrangement between the Humboldt Institute in Villa de Leyva, Colombia, the University of Kentucky, and the Natural History Museum of Los Angeles County (LACM) – funded by U. S. National Science Foundation (NSF DEB 9972024) and the Humboldt Institute (see also http://www.sharkeylab.org/biodiversity/static.php?app=colombia&page=index). The material herein studied was collected from three protected areas of Colombia (see also http://www.parquesnacionales.gov.co/ and Fig. 38) as follows: 1) the “Parque Nacional Natural Farralones de Cali” (“PNN Farralones de Cali” within the label data) located on the West Cordillera and characterized by a great variety of climates that are reflected in a variety of ecosystems, as cold regions with paramillos and its diverse vegetation, warm areas with plants with tabular roots and reaching considerable heights, and temperate areas with oaks and black oaks; 2) the “Santuario de Flora y Fauna Otún Quimbaya” (“SFF Otún Quimbaya“ within the label data) characterized by evergreen sub-Andean jungle vegetation, in which the effect of the rainy and dry seasons is masked by the usual presence of mist formations and there is gradual replacement of low synchrony in the production of fruits and leaves, and is located on the West Cordillera, and 3) the “Santuario de Flora y Fauna Iguaque” (“SFF Iguaque” within the label data) locating on the East Cordillera, in a region of paramo and Andean forest ecosystems, including a representative sample of oak forest.

The examined material was initially stored in ethyl alcohol, within which most specimens – after study under a stereomicroscope Leica S8APO – are still preserved. In case of several specimens, for more detailed study of male terminalia, they were detached and macerated in a solution of KOH, followed by neutralization in acetic acid and washing in distilled water. The remaining chitinous parts were thereafter inserted into glycerine for study including illustrations and preserved as glycerine preparations in polyethylene microvials (cf. Kurina 2003). A few specimens including their terminalia were slide mounted in Euparal following the method described by Hippa and Kurina (2012). The preservation method of each specimen is indicated in the material section. The measurements are given as the range of measured specimens followed by the mean value, while the measurements and setosity information from the holotype are given in square brackets. The ratios of the three apical palpal segments are given as 3^rd^:4^th^:5^th^. All measurements are taken from specimens in alcohol. Morphological terminology follows generally that of Søli et al. (2000) and Amorim and Rindal (2007) while the interpretation by Kjærandsen (2006) and Oliveira and Amorim (2012) are used for terminalia and thorax, respectively.

The habitus photos have been made in an alcohol medium and combined by software LAS V.4.5.0. from multiple gradually focused images taken by a camera Leica DFC 450 attached to the stereomicroscope Leica M205C. The photos of terminalia were combined by the same software but the camera was attached to the compound microscope Leica DM 6000 B (see also Kurina et al. 2015). Adobe Photoshop CS5 was used for editing the figures and compiling the plates.

The distributional map was performed with the software DIVA-GIS 7.5.0 (http://www.diva-gis.org/) and edited with Adobe Photoshop. The shapefile maps from Colombia and South America were obtained from DIVA-GIS and Dreamstime (ID 10514087 © Michael Schmeling and Dreamstime.com) websites, respectively.

The following acronyms are used for depositories:

IAvH Instituto de Investigación de Recursos Biológicos Alexander von Humboldt, Villa de Leyva, Boyacá, Colombia.

IZBE Institute of Agricultural and Environmental Sciences, Estonian University of Life Sciences [former Institute of Zoology and Botany], Tartu, Estonia.

MZUSP Museu de Zoologia da Universidade de São Paulo, São Paulo, Brazil.

## The species

### Cordyla monticola sp. n.

Taxon classificationAnimaliaDipteraMycetophilidae

http://zoobank.org/7A0180A6-F306-4E8D-B566-89853152F6AA

Figs 1–2 , 3–7 , 8–9 , 38

#### Type material.

*Holotype.* ♂, COLOMBIA, Boyacá / SFF Iguaque El Níspero / 05°38'N 73°31'W 2730 m / Malaise 2 07-21.xii.2001 / P. Reina Leg. M. 2585 [IAvH]. *Paratype.* 1♂, same as holotype [MZUSP].

#### Description.

**Figures 1–2.**
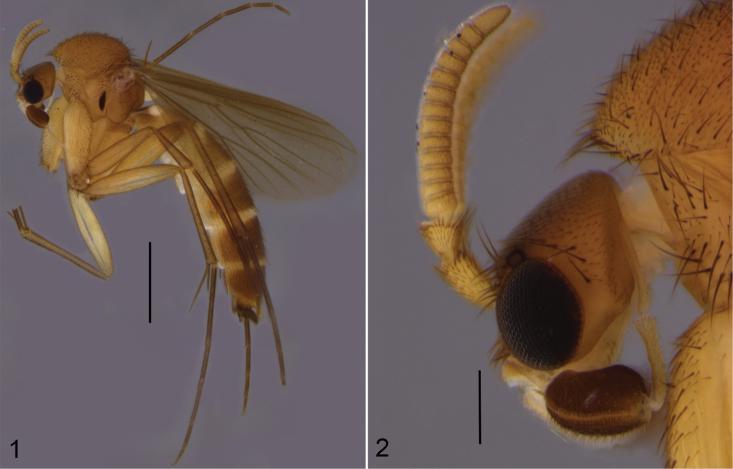
*Cordyla monticola* sp. n. **1** male habitus **2** head with antennae and maxillary palpi, closer view. Scale bars: 1 mm (**1**) and 0.2 mm (**2**).

**Male** (Fig. 1). Total length 4.1–4.6, 4.4 [4.6] mm (n=2).

**Figures 3–7.**
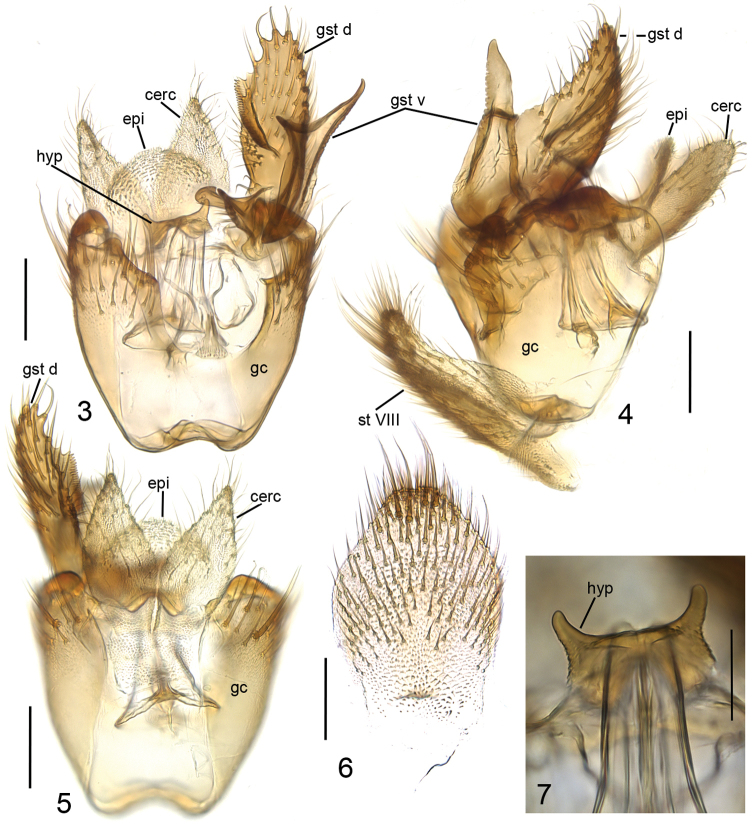
*Cordyla monticola* sp. n., male terminalia. **3** ventral view **4** lateral view **5** dorsal view **6** sternite VIII, ventral view **7** hypoproct, ventral view. Scale bars: 0.1 mm (**3, 4, 5, 6**) and 0.05 mm (**7**). Abbreviations: cerc = cercus; epi = epiproct; gc = gonocoxite; gst d = dorsal branch of gonostylus; gst v = ventral branch of gonostylus; hyp = hypoproct; st VIII = sternite VIII.

**Figures 8–9.**
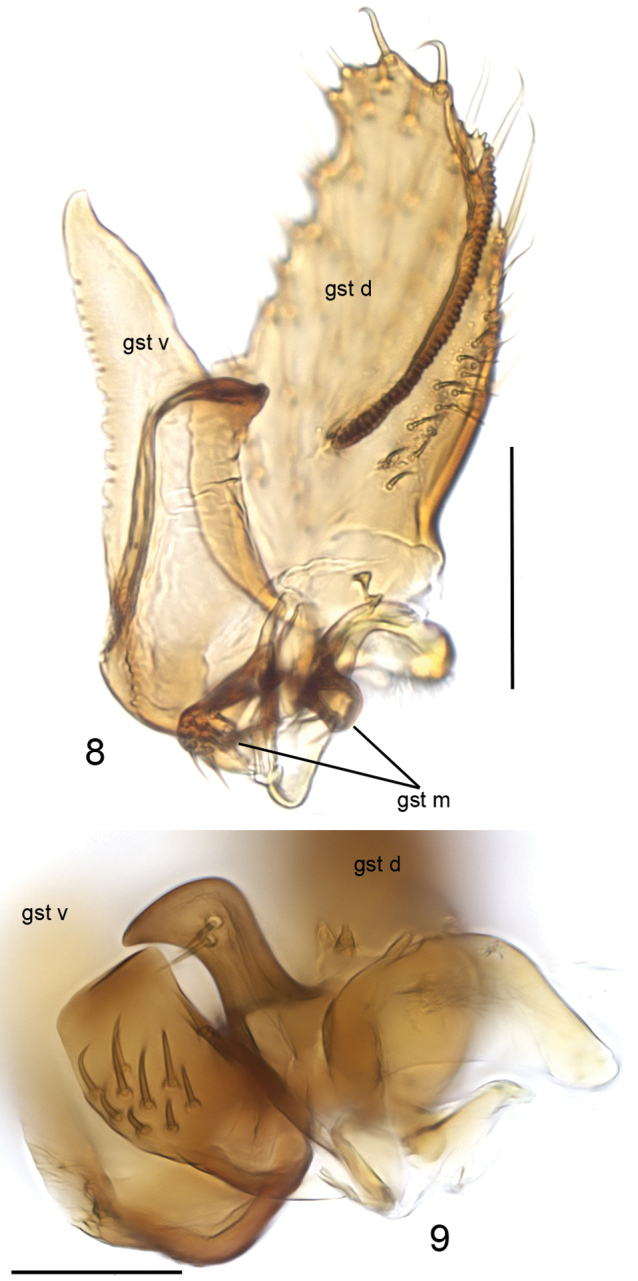
*Cordyla monticola* sp. n., gonostylus. **8** internal view **9** lobes of medial branch of gonostylus. Scale bars: 0.1 mm (**8**) and 0.05 (**9**). Abbreviations: gst d = dorsal branch of gonostylus; gst m = medial branch of gonostylus; gst v = ventral branch of gonostylus.

**Head** (Fig. 2) dark yellow, mouthparts pale. Two ocelli encircled by brown areas, close to compound eyes. All three visible palpal segments (Fig. 2) setose, swollen antepenultimate segment dark brown, succeeding segments pale. 4^th^ segment slightly widening apically, 5^th^ segment apically tapering. Swollen palpal segment 1.6 times as long as broad medially from lateral view, and 1.1–1.3, 1.2 [1.1] times as long as height of compound eye. Ratios of three apical palpomeres 1.0: 0.9: 0.9. Antenna yellow with 2+13 segments. Scape and pedicel with brown setae, flagellum with somewhat paler setosity. Scape elongate cup-shaped, 1.7–2.2, 2.0 [2.2] times as long as wide apically. Pedicel cup-shaped, 0.9 times as long as wide apically. Flagellomeres rectangular, about twice as wide as long. Apical flagellomere conical, about 1.6 times as long as wide basally. **Thorax** yellow, mesonotum medially, laterotergite and mediotergite somewhat darker. Hind margin of laterotergite narrowly brown. Anterior part of mesepimeron with a dark brown patch leaving anteroapical margin yellow. Haltere with pale knob, and basally pale and apically yellow stem. All setosity on thorax brown. Scutum entirely covered with decumbent setae, scutellum with setae including two pairs of marginal bristles, laterals shorter than internals. Antepronotum with setae including 6–8 [8] bristles, proepisternum with setae including 6–8 [8] bristles. Anepisternum with 2–4 [4] bristles at hind margin and with ca. 50 setae on its upper two thirds. Mesepimeron and katepisternum bare. Laterotergite with 5–6 [6] bristles and ca. 20 setae on upper half. Mediotergite bare. Metepisternum with 5–7 [7] bristles and ca. 10 setae. **Wing** with yellowish tinge, cell R1 somewhat darker. Length 3.1 [3.1] mm (n=2). Ratio of length to width 2.5–2.6, 2.6 [2.6]. All veins light brown. Radial veins seem darker because of setae on both surfaces; other veins bare. Crossvein r-m apically disjunct. M1+2 3.5–3.7, 3.6 [3.7] times as long as r-m. M2 not reaching wing margin, broken 0.4–0.8, 0.6 [0.4] times of M1+2 length before it. Posterior fork begins clearly before anterior fork, at the middle of M1+2. **Legs** yellow, hind femora infuscated at apical fourth. Tarsi seem darker because of dense brown setae. Hind coxa with 4 [4] posterolateral bristles basally, with 0–2 [2] posterior bristles apically, and with ca. 30 weaker setae along posterolateral margin. Ratio of femur to tibia for fore-, mid- and hind legs: 1.5; 1.1; 1.0. Ratio tibia to first tarsomere for fore-, mid- and hind legs: 1.0; 1.2; 1.4. Fore-tibia with a spur about 0.5 of fore basitarsus; mid-tibia with anterior spur about 0.3–0.4, 0.4 [0.3] and with posterior spur about 0.6–0.7, 0.7 [0.6] of mid basitarsus; hind tibia with anterior spur about 0.5–0.6, 0.6 [0.5] and with posterior spur about 0.6–0.7, 0.7 [0.6] of hind basitarsus. **Abdomen** with first segment dorsally and laterally light brown and ventrally yellow. 2–4 segments dorsally brown with anterior and posterior margins yellowish, and laterally and ventrally yellowish; succeeding segments brownish. **Terminalia** (Figs 3–9) with gonocoxite basally yellow and apically brownish; gonostylus brownish; sternite 8 seems brownish because of dense setosity. Basal two thirds of sternite 8 cylindrical, apical third tapering, apex truncated. Basal third of sternite 8 membranous and bare, apical setae stronger than other setae. Gonocoxite slightly oblong, with broad ventral incision more than half of gonocoxite height. Ventral incision of gonocoxite with apically pointed basal projection about one third height of incision. Ventral medial margin of gonocoxite angular. Dorsal medial margin of gonocoxite simple. Cerci setose, clearly separated, basally wide, well tapering apically and protruding over gonocoxite. Basal half of gonocoxite bare, apical half with strong bristles. Dorsal branch of gonostylus rectangular, apically rounded, with a medially situated sclerotized comb of about half height of branch on its ventral surface. Apical setae somewhat stronger, deviating from other setosity of the branch. Dorsal branch of gonostylus with an indistinct basal tubercle on its ventral surface, close to base of medial branch. Ventral branch of gonostylus bare, subequal to dorsal branch, with serrated lateral margin and medially drawn out to a distinct lobe. The apical third of ventral branch is well tapering in ventral view. Medial branch of gonostylus divided at apical two thirds into two subequal lobes: ventral lobe rectangular, apically truncated, bearing 9-10 setae on its ventral part; dorsal lobe beak-shaped with two setae subapically on its ventral margin. Epiproct rounded with small setulae that arise in lines of 4 to 8 from small ridges. Hypoproct bowl-shaped with well-outlined lateral shoulders.

**Female.** Unknown.

#### Biology.

Unknown.

#### Etymology.

The species is named to indicate its occurrence at high altitude (2730 m a.s.l.): Latin *monticola* means “mountain dweller”. The specific epithet is noun in apposition.

#### Comments.

The paratype has seemingly 14 flagellar segments at one side, caused by an aberrantly divided apical one. This, as well as partial fusion of some flagellar segments unilaterally, is common and frequently observed in the Palaearctic specimens of the genus (OK *pers. obs.*). According to the structure of male terminalia, especially in having the medial branch of the gonostylus divided into two subequal lobes, the species belongs to the *Cordyla murina* species-group as defined by Kurina (2001). Within the group, *Cordyla monticola* sp. n. shares a 13-segmented flagellum and brown to dark brown swollen antepenultimate palpal segment with two Palaearctic (viz. *Cordyla semiflava* Staeger, 1840 and *Cordyla borealisa* Wu in Wu & Zheng, 2000) and three Nearctic (*Cordyla manca* Johannsen, 1912, *Cordyla scita* Johannsen, 1912 and *Cordyla gracilis* Fisher, 1938) species. *Cordyla semiflava*, *Cordyla borealisa* and *Cordyla manca* have the sternite VIII subapically remarkably constricted, while it is smoothly tapering in *Cordyla monticola* sp. n. The shape of the lobes of medial branch of the gonostylus and the hypoproct are different from those in all species of the group.

### Cordyla pseudopusilla sp. n.

Taxon classificationAnimaliaDipteraMycetophilidae

http://zoobank.org/1EF47BC4-2CCE-449D-B9D7-95165BBC79FA

Figs 10–11 , 12–16 , 17–19 , 20–23 , 38

#### Type material.

*Holotype.* ♂, COLOMBIA Boyacá / SFF Iguaque El Níspero / 05°38'N 73°31'W 2730 m / Malaise 1 13-28.x.2001 / P. Reina Leg. M. 2475 [IAvH]. *Paratypes.* 1♂, same as holotype except 28.x-14.xi.2001, M. 2482 [IAvH]; 1♂ 2♀♀, same as holotype except 2 07-21.xii.2001, M. 2585 [1♂ 1♀ at MZUSP, 1♀ at IAvH]; 1♂ 1♀, same as holotype except 19.i-03.ii.2002, M. 3067 [IZBE]; 1♂, same as holotype except 3-18.ii.2002, M. 3068 [IAvH].

#### Description.

**Figures 10–11.**
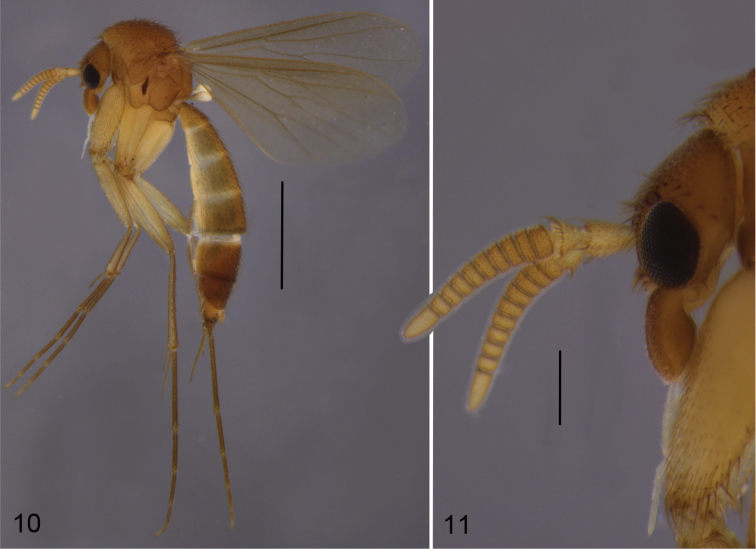
*Cordyla pseudopusilla* sp. n. **10** male habitus **11** head with antennae and maxillary palpi, closer view. Scale bars: 1 mm (**10**) and 0.2 mm (**11**).

**Male** (Fig. 10). Total length 2.9–3.4, 3.2 [3.4] mm (n=5).

**Figures 12–16.**
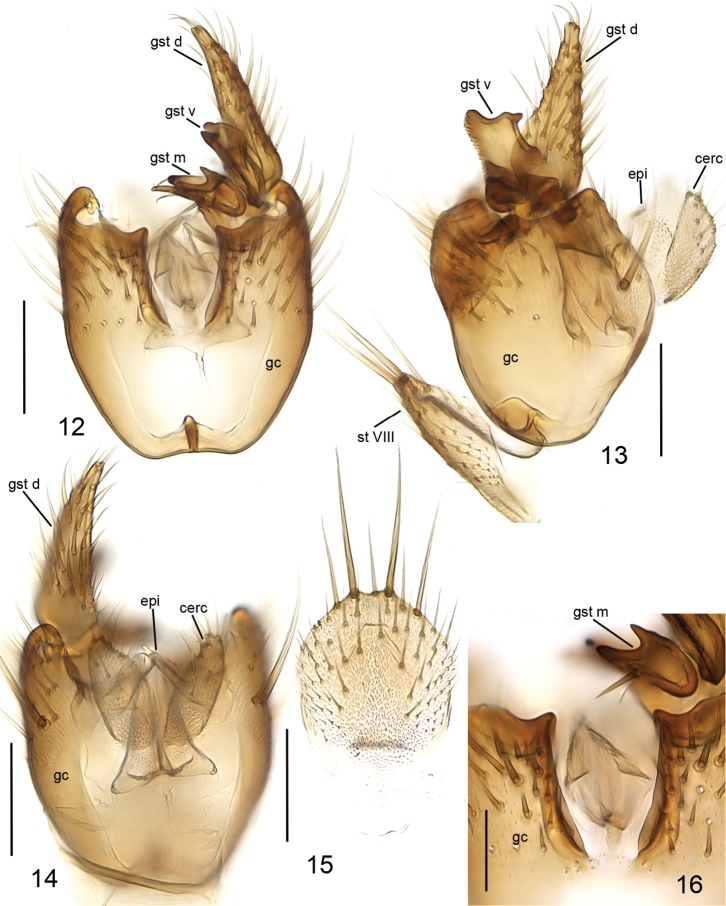
*Cordyla pseudopusilla* sp. n., male terminalia. **12** ventral view **13** lateral view **14** dorsal view **15** sternite VIII, ventral view **16** ventromedial incision of gonocoxite, ventral view. Scale bars: 0.1 mm (**12, 13, 14, 15**) and 0.05 mm (**16**). Abbreviations: cerc = cercus; epi = epiproct; gc = gonocoxite; gst d = dorsal branch of gonostylus; gst m = medial branch of gonostylus; gst v = ventral branch of gonostylus; st VIII = sternite VIII.

**Figures 17–19.**
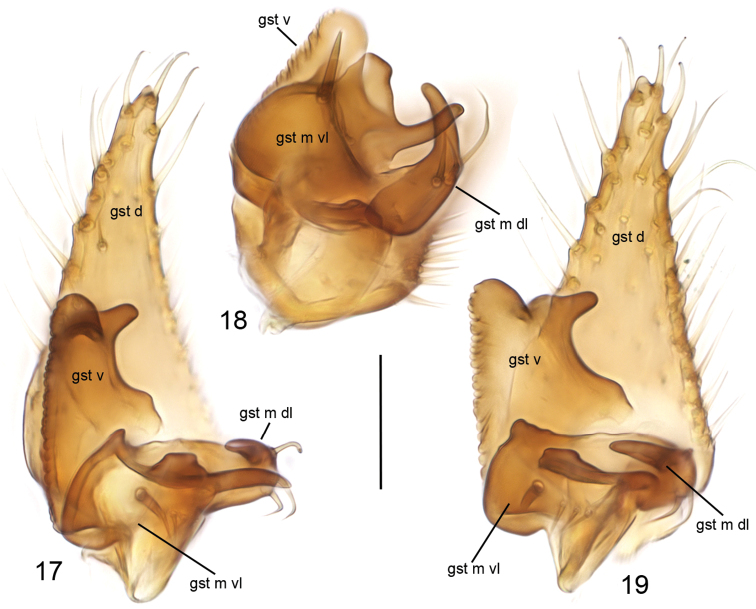
*Cordyla pseudopusilla* sp. n., gonostylus **17** ventral view **18** anterior view of medial branch **19** internal view. Scale bars: 0.05 mm. Abbreviations: gst d = dorsal branch of gonostylus; gst m dl = dorsal lobe of medial branch of gonostylus; gst m vl = ventral lobe of medial branch of gonostylus; gst v = ventral branch of gonostylus.

**Head** (Fig. 11) dark yellow, mouthparts somewhat paler. Two ocelli encircled by brown areas, close to compound eyes. All three visible palpal segments (Fig. 11) setose, swollen antepenultimate segment light brown to brown, succeeding segments pale. 4^th^ segment slightly widening apically, 5^th^ segment apically tapering. Swollen palpal segment 1.8–2.2, 2.0 [1.8] times as long as broad medially from lateral view, and as long as height of compound eye. Ratios of three apical palpomeres 1.0: 0.8: 1.0. Antenna with 2+10 segments. Scape and pedicel yellow, flagellum somewhat darker, all with brown setosity. Scape elongate cup-shaped, 1.9–2.3, 2.1 [2.0] times as long as wide apically. Pedicel cup-shaped, 0.6–0.8, 0.7 [0.6] times as long as wide apically. Flagellomeres rectangular, about twice as wide as long. Apical flagellomere conical, about 2.2 times as long as wide basally. **Thorax** yellow, mesonotum medially somewhat darker. Hind margin of laterotergite narrowly brown. Katepisternum lighter. Anterior part of mesepimeron with a brown patch leaving anteroapical corner yellow. Haltere with a pale knob, stem basally pale and darker. All setosity on thorax brown. Scutum entirely covered with decumbent setae, scutellum with setae including two pairs of marginal bristles, laterals considerably shorter than internals. Antepronotum with setae including 5–7 [7] bristles, proepisternum with setae including 4–7 [7] bristles. Anepisternum with 4 bristles at hind margin and with ca. 40 setae on its upper two thirds. Mesepimeron and katepisternum bare. Laterotergite with 2–3 [3] bristles medially and ca. 10 setae along upper margin. Mediotergite bare. Metepisternum with 5–6 [5] bristles and ca. 10 setae. **Wing** with yellowish tinge, otherwise clear. Length 2.2–2.5, 2.4 [2.4] mm (n=10). Ratio of length to width 2.4–2.7, 2.6 [2.4]. All veins light brown. Radial veins seem darker because of setae on both surfaces; other veins bare. Crossvein r-m apically disjunct. M1+2 5–6 times as long as r-m. M2 not reaching wing margin, broken 0.5–0.7, 0.6 [0.5] times of M1+2 length before it. M1 and M4 apically very faint. Posterior fork begins clearly before anterior fork. **Legs** yellow, hind femora slightly infuscated at apical fifth. Tarsi seem darker because of dense brown setae. Hind coxa with 2–3 [3] posterolateral bristles basally, with one posterior bristle apically, and with ca. 10 weaker setae along posterolateral margin. Ratio of femur to tibia for fore-, mid- and hind legs: 1.5–1.6, 1.6 [1.5]; 1.0–1.1, 1.1 [1.1]; 1.0, 1.0 [1.0]. Ratio tibia to first tarsomere for fore-, mid- and hind legs: 1.0–1.1, 1.0 [1.0]; 1.1–1.3, 1.2 [1.1]; 1.4–1.5, 1.5 [1.4]. Fore-tibia with a spur about 0.5–0.6, 0.5 [0.6] of fore basitarsus; mid-tibia with anterior spur about 0.3, 0.3 [0.3] and with posterior spur about 0.6–0.7, 0.7 [0.7] of mid basitarsus; hind tibia with anterior spur about 0.5 and with posterior spur about 0.6 of hind basitarsus. **Abdomen** with first segment light brown, and 2–4 segments dorsally brownish, laterally yellow and ventrally pale yellow; succeeding segments entirely brown. **Terminalia** (Figs 12–19) with basal part of gonocoxite and cerci yellow; apical part of gonocoxite slightly darker; dorsal branch of gonostylus yellow; ventral and medial branches of gonostylus brown. Sternite 8 oblong, apical fourth conical, apex truncated, basal half membranous and bare, setae on apical quarter somewhat stronger than other setae, two apical setae well deviating from other setosity. Gonocoxite subquadrate, with narrow ventral incision about half height of gonocoxite. Ventral medial margin of gonocoxite apically angular and with membranous formations dorsad from the ventral surface of gonocoxite. Dorsal medial margin of gonocoxite simple. Cerci setose, setae on medial margin slightly stronger, deviating from other setosity; clearly separated, prolonged, subapically somewhat constricted, not protruding over gonocoxite. Basal half of gonocoxite bare, apical half with strong bristles. Dorsal branch of gonostylus elongated, tapering, without sclerotized comb, dorsal surface with homogeneous setosity. Ventral branch of gonostylus bare, about half as long as dorsal branch, with serrated lateral margin. Apical corners of ventral branch of gonostylus drawn out to small lobes: ventral wider and rounded, dorsal narrow. Dorsal margin of ventral branch of gonostylus with wide and shallow incision. Medial branch of gonostylus divided into two lobes: 1) ventral lobe with three apical protrusions separated by concavities (ventral finger-like protrusion well discernible at internal view), internal surface with one strong and three weaker setae, and 2) dorsal lobe curved, tapering, as long as the medial protrusion of ventral lobe and with three medial setae. Epiproct apically rounded, covered with small setulae. Hypoproct indiscernible.

**Figures 20–23.**
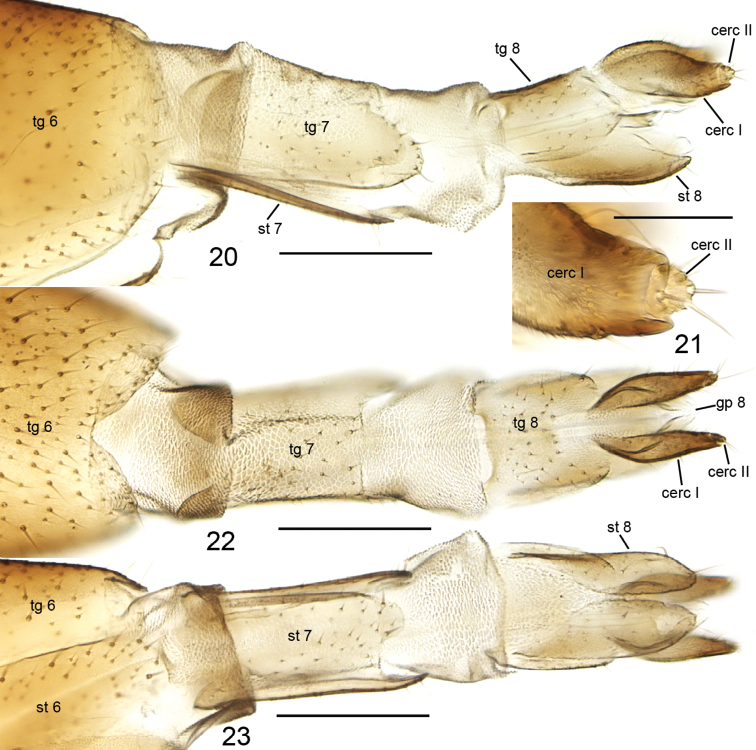
*Cordyla pseudopusilla* sp. n. female terminalia. **20** lateral view **21** cerci, closer view **22** dorsal view **23** ventral view. Scale bars: 0.2 mm (**20, 22, 23**) and 0.05 mm (**21**). Abbreviations: cerc = cercus, gp = gonapophysis, st = sternite, tg = tergite.

**Female.** Total length 3.6–3.7, 3.6 mm (n=3). Wing length 2.4–2.5, 2.4 mm. Ratio of length to width 2.4–2.6, 2.5. Antennae 2+9 segments. In setosity and coloration similar to male. Terminalia (Figs 20–23) light brown. Cercus two-segmented: apical segment small, sunken into basal segment, with 2-3 long setae deviating from other setosity; basal segment long ovate, slightly sinusoidal and considerably wider than apical segment. Gonapophysis VIII membranous, visible in dorsal view. Tergite VIII rectangular, subequal to length of basal segment of cercus, apically angular in lateral view, basally and apically well emarginated in dorsal view. Sternite VIII tapering lateroapically, with deep medial cleft in ventral view. Tergite VII longer than tergite VIII, basally and apically well emarginated in dorsal view. Sternite VII apically conical, subequal to length of tergite VII. Tergite VI apically rounded in lateral view and with apicodorsal incision.

#### Biology.

Unknown.

#### Etymology.

The specific name is derived by the Greek prefix *pseudo*– from the Palaearctic species *Cordyla pusilla* Edwards, 1925 to indicate their morphological similarity.

#### Comments.

Belonging to the *Cordyla fusca* species-group (cf. Kurina 2001), *Cordyla pseudopusilla* sp. n. shares a 10-segmented flagellum and brown to dark brown swollen antepenultimate segment of palpus with five Palaearctic (viz. *Cordyla bomloensis* Kjærandsen & Kurina, 2004, *Cordyla brevicornis* Staeger, 1840, *Cordyla geminata* Sasakawa, 2005, *Cordyla pusilla* and *Cordyla triloba* Sasakawa, 2008) and three Nearctic (viz. *Cordyla neglecta* Johannsen, 1912, *Cordyla recens* Johannsen, 1912, *Cordyla scutellata* Garrett, 1925) species. Within these, the new species is remarkably similar to *Cordyla pusilla* and *Cordyla neglecta*. All three species have the ventral branch of gonostylus subquadrate with emarginated dorsal margin. However, the emargination is more pronounced in *Cordyla pseudopusilla* sp. n. The new species has the medial branch of the gonostylus with a curved dorsal lobe (straight in the other two species) and with deep concavities on the ventral lobe apically (more shallow in the other two species).

### Cordyla reducta sp. n.

Taxon classificationAnimaliaDipteraMycetophilidae

http://zoobank.org/8FBF8F6C-601E-4CE3-80D7-39E72B2108C5

Figs 24–25 , 26 , 27–31 , 32–33 , 34–37 , 38

#### Type material.

*Holotype.* ♂, COLOMBIA, Risaralda SFF / Otún Quimbaya Robledal / 04°44'N 75°35'W 1980 m /Malaise 04–20.iii.2003 / G. López Leg. M. 3686 [IAvH]. *Paratypes.* 1♂ 2♀♀, same as holotype [IAvH]; 1♂, Boyacá / SFF Iguaque El Níspero / 05°38'N 73°31'W 2730 m / Malaise 2 07-21.xii.2001 / P. Reina Leg. M. 2585 [IAvH]; 4♂♂ 2♀♀, Valle del Cauca / PNN Farallones de Cali / Cgto. La Meseta / 03°34'N 76°40'W 1960 m / Malaise 09-26.x.2003 / S. Sarria & M. Losso Leg. M. 4548 [IZBE]; 1♂, Valle del Cauca / PNN Farallones de Cali / Cgto. La Meseta / 03°34'N 76°40'W 1960 m / Malaise 27.viii-10.ix.2003 / S. Sarria & M. Losso Leg. M. 4549 [IAvH]; 1♂ 2♀♀, Valle de Cauca / PNN Farallones de Cali Cgto. / La Meseta 03°34'N 76°40'W 2200 m / Malaise 26.xi-10.xii.2003 / S. Sarria & M. Losso Leg. M. 4562 [MZUSP]; 2♂♂, COLOMBIA, Risaralda SFF / Otún Quimbaya Robledal / 04°44'N 75°35'W 1980 m Malaise 20.iii-04.iv.2003 / G. López Leg. M. 3682 [MZUSP]; 1♀, Risaralda SFF / Otún Quimbaya Urapanera / 04°44'N 75°35'W 1960 m Malaise 20.iii–04.iv.2003 / G. López Leg. M. 3688 [IAvH]; 1♂ 3♀♀, Risaralda SFF / Otún Quimbaya Robledal / 04°44'N 75°35'W 1980 m Malaise 18.ii-04.iii.2003 / G. López Leg. M. 3699 [IAvH]; 1♀, Risaralda SFF / Otún Quimbaya Robledal / 04°44'N 75°35'W 1980 m Malaise 04-19.iv.2003 / G. López Leg. M. 3710 [IAvH]; 5♂♂ 3♀♀ COLOMBIA, Risaralda SFF / Otún Quimbaya Robledal / 04°44'N 75°35'W 1980 m Malaise 11-27.x.2003 / G. López Leg. M. 4182 [3♂♂ 2♀♀ MZUSP, 2♂♂ 1♀ IZBE].

#### Description.

**Figures 24–25.**
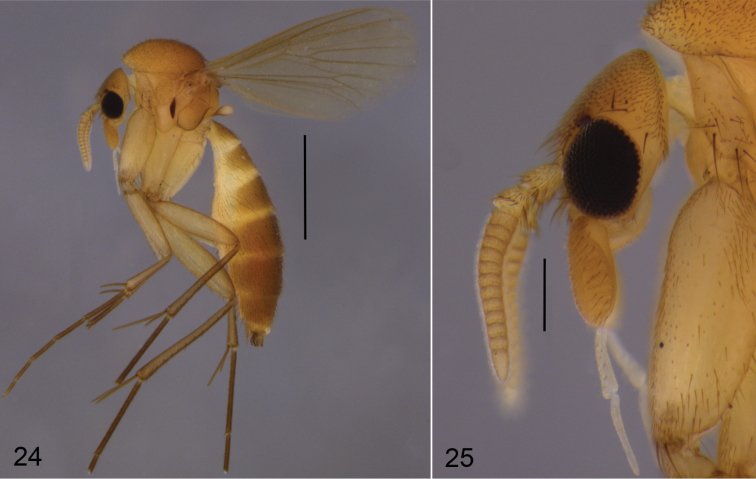
*Cordyla reducta* sp. n. **24** male habitus **25** head with antennae and maxillary palpi, closer view. Scale bars: 1 mm (**24**) and 0.2 mm (**25**).

**Male** (Fig. 24). Total length 2.5–3.2, 2.9 [3.2] mm (n=10).

**Figure 26.**
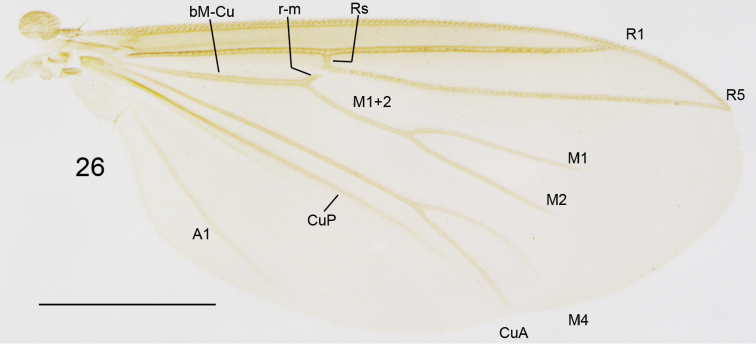
*Cordyla reducta* sp. n. **26** female wing. Scale bars: 0.5 mm.

**Figures 27–31.**
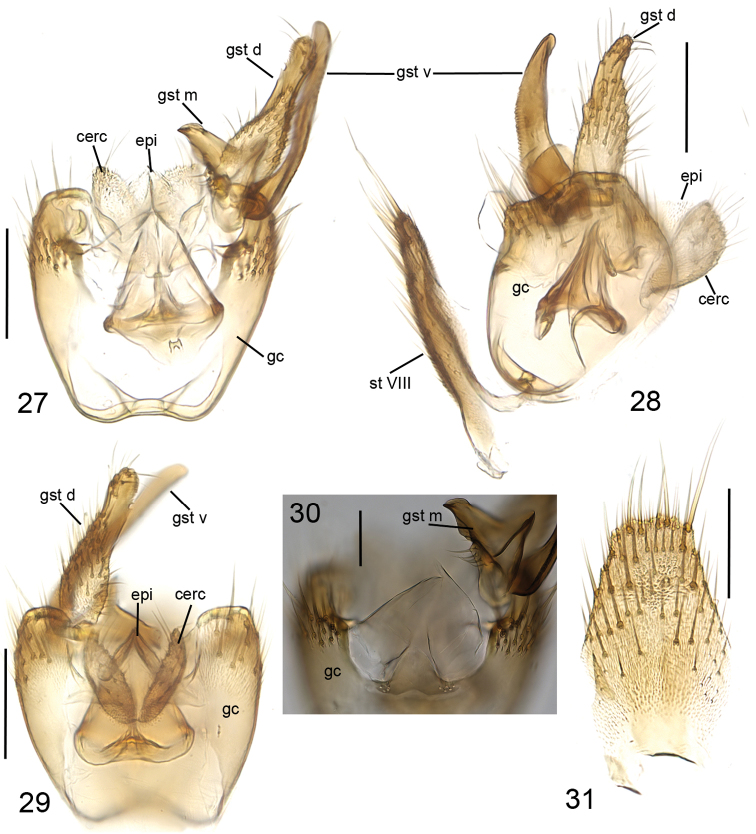
*Cordyla reducta* sp. n., male terminalia. **27** ventral view **28** lateral view **29** dorsal view **30** ventromedial incision of gonocoxite, ventral view **31** sternite VIII, ventral view. Scale bars: 0.1 mm (**27, 28, 29, 31**) and 0.05 mm (**30**). Abbreviations: cerc = cercus; epi = epiproct; gc = gonocoxite; gst d = dorsal branch of gonostylus; gst m = medial branch of gonostylus; gst v = ventral branch of gonostylus; st VIII = sternite VIII.

**Figures 32–33.**
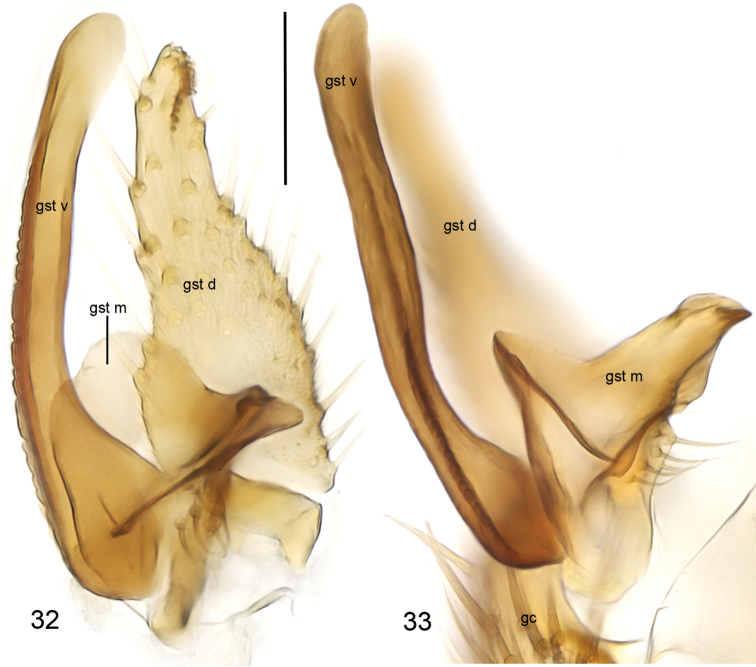
*Cordyla reducta* sp. n., gonostylus **32** internal view **33** ventral view. Scale bars: 0.05 mm. Abbreviations: gc = gonocoxite; gst d = dorsal branch of gonostylus; gst m = medial branch of gonostylus; gst v = ventral branch of gonostylus.

**Head** (Fig. 25) dark yellow, mouthparts somewhat paler. Two ocelli encircled by brown areas, close to compound eyes. All three visible palpal segments (Fig. 25) setose, swollen antepenultimate segment dark yellow, succeeding segments pale. 4^th^ segment slightly widening apically, 5^th^ segment apically tapering. Swollen palpal segment 2.3–2.6, 2.4 [2.5] times as long as broad medially from lateral view, and 1.1–1.3, 1.2 [1.1] times as long as height of compound eye. Ratios of three apical palpomeres 1.0: 0.7: 1.0–1.1, 1.0 [1.1]. Antenna yellow with 2+12 segments. Scape and pedicel with brown setae, flagellum with somewhat paler setosity. Scape elongate cup-shaped, 1.8–2.2, 2.0 [1.9] times as long as wide apically. Pedicel cup-shaped, 0.7–0.9, 0.8 [0.9] times as long as wide apically. Flagellomeres rectangular, about twice as wide as long. Apical flagellomere conical, about 1.7 times as long as wide basally. **Thorax** yellow, mesonotum medially somewhat darker. Hind margin of laterotergite narrowly brown. Anterior part of mesepimeron with a brown patch. Haltere with knob apically pale and basally brownish, and pale stem. All setosity on thorax brown. Scutum entirely covered with decumbent setae, scutellum with setae including two pairs of marginal bristles, laterals considerably shorter than internals. Antepronotum with setae including 2–3 [3] bristles, proepisternum with setae including 3–5 [5] bristles. Anepisternum with 3–4 [4] bristles at hind margin and with ca. 45 setae on its upper two thirds. Mesepimeron and katepisternum bare. Laterotergite with 2 bristles and ca. 15 setae on upper half. Mediotergite bare. Metepisternum with 2–4 [4] bristles and ca. 10 setae. **Wing** with yellowish tinge, otherwise clear. Length 2.0–2.3, 2.2 [2.3] mm (n=10). Ratio of length to width 2.6–2.9, 2.7 [2.9]. All veins light brown. Radial veins seem darker because of setae on both surfaces; other veins bare, except about 5 setae on dorsal surface of M4. Crossvein r-m apically disjunct. M1+2 3–4 times as long as r-m. R5 slightly sinusoid. M2 not reaching wing margin, broken 0.7–1.0, 0.8 [0.9] times of M1+2 length before it. Posterior fork begins clearly beyond anterior fork. **Legs** yellow, hind femora slightly infuscated at apical fifth. Tarsi seem darker because of dense brown setae. Hind coxa with 2–4 [4] posterolateral bristles basally, with one posterior bristle apically, and with ca. 25 weaker setae along posterolateral margin. Ratio of femur to tibia for fore-, mid- and hind legs: 1.3–1.6, 1.4 [1.4]; 0.8–1.0, 0.9 [1.0]; 1.0, 1.0 [1.0]. Ratio tibia to first tarsomere for fore-, mid- and hind legs: 1.0–1.1, 1.0 [1.0]; 1.1–1.3, 1.2 [1.2]; 1.4, 1.4 [1.4]. Fore-tibia with a spur about 0.5–0.6, 0.5 [0.6] of fore basitarsus; mid-tibia with anterior spur about 0.3 and with posterior spur about 0.6–0.7, 0.7 [0.6] of mid basitarsus; hind tibia with anterior spur about 0.6 and with posterior spur about 0.6–0.7, 0.7 [0.6] of hind basitarsus. **Abdomen** with first 3 segments dorsally brownish, laterally yellow and ventrally pale yellow; succeeding segments brown, only slightly lighter laterally and ventrally. **Terminalia** (Figs 27–33) with gonocoxite and cerci yellow; gonostylus brownish; sternite 8 seems brownish because of dense setosity. Sternite 8 oblong with truncated apex, basal third membranous and bare, setae on apical quarter somewhat stronger than rest of them. Gonocoxite subquadrate, with broad ventral incision about half of gonocoxite height. Ventral incision of gonocoxite with apically rounded basal hump about one third height of incision. Ventral medial margin of gonocoxite angular with apically pointed membranous formations dorsad from the ventral surface of gonocoxite. Dorsal medial margin of gonocoxite bulging. Cerci setose, setae on medial margin stronger, deviating from other setosity; clearly separated, prolonged, subapically somewhat constricted, not protruding over gonocoxite. Basal half of gonocoxite bare, apical half with strong bristles. Dorsal branch of gonostylus oblong, basally wider, apically slightly tapering, with a sclerotized comb of about one fifth branch height on its ventral surface. Setosity on basal two thirds more dense, leaving subapical area almost bare; apical setae slightly stronger than other setosity of the branch. Ventral branch of gonostylus bare, basally bulbous and apically rounded, slightly curved, longer than dorsal branch, with serrated lateral margin. Medial branch of gonostylus hump-backed, not divided into lobes but medially extended, and with 5–6 strong setae at medial margin ventrobasally. Epiproct abruptly narrowing subapically, with pointed apex, covered with small setulae. Hypoproct indiscernible.

**Figures 34–37.**
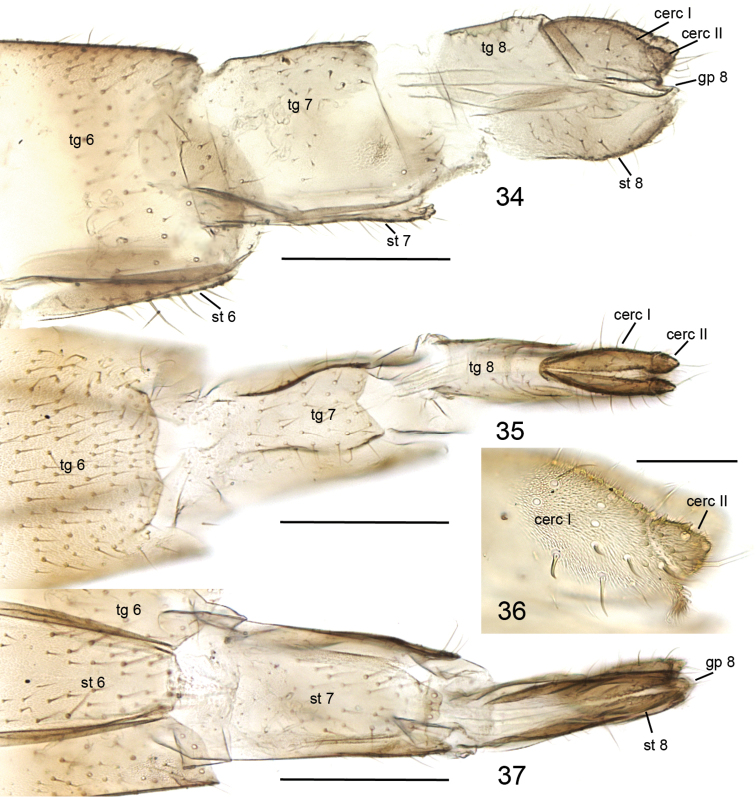
*Cordyla reducta* sp. n. female terminalia. **34** lateral view **35** dorsal view **36** cerci, closer view **37** ventral view. Scale bars: 0.2 mm (**34, 35, 37**) and 0.05 mm (**36**). Abbreviations: cerc = cercus, gp = gonapophysis, st = sternite, tg = tergite.

**Female.** Total length 2.5–3.4, 2.9 mm. Wing length 1.7–2.5, 2.1 mm. Ratio of length to width 2.4–2.7, 2.6. Antennae 2+9 segments. In setosity and coloration similar to male, except for entirely pale second abdominal segment. Both M1 and M2 not reaching wing margin, M4 extremely faint at its distal part (Fig. 26). Terminalia (Figs 34–37) light brown. Cercus two-segmented: apical segment small, with a few long setae deviating from other setosity, obliquely connected with basal segment; basal segment long ovate, sinusoidal and wider than apical segment, ventroapical corner drawn out to a setose small lobe. Gonapophysis VIII membranous, visible in ventral view, apically rounded. Tergite VIII rectangular, subequal to length of basal segment of cercus, apically angular, basally and apically emarginated in dorsal view. Sternite VIII tapering lateroapically, with deep medial cleft in ventral view. Tergite VII about twice as long as tergite VIII, apically widening and with apical incision in dorsal view. Sternite VII apically conical, subequal to length of tergite VII. Tergite VI apically emarginated in dorsal view.

#### Biology.

Unknown.

#### Etymology.

The specific name refers to the distally extremely reduced medial veins of the female wing: from Latin *reducta* meaning “distant”. An adjective.

#### Comments.

By the structure of male terminalia, *Cordyla reducta* sp. n. belongs to the *Cordyla fusca* species-group (cf. Kurina 2001). In its 12-segmented flagellum and yellow to light brown swollen antepenultimate segment of palpus, the new species is similar to the Palaearctic *Cordyla flaviceps* (Staeger, 1840) and Oriental *Cordyla borneoensis* Kurina, 2005. The male terminalia of *Cordyla reducta* sp. n. are remarkably different from these two species but otherwise resemble the Palaearctic *Cordyla fasciata* Meigen, 1818 in having a similar outline to the ventral and dorsal branches of the gonostylus. However, the medial branch of the gonostylus is hump-backed and medially extended in *Cordyla reducta* sp. n., while it has two small medial lobes in *Cordyla fasciata* (cf. Zaitzev 2003: fig. 21–27). All studied female specimens have both medial veins of the wing distally extraordinary reduced (Fig. 26), unique among the species worldwide.

## Discussion

**Figure 38.**
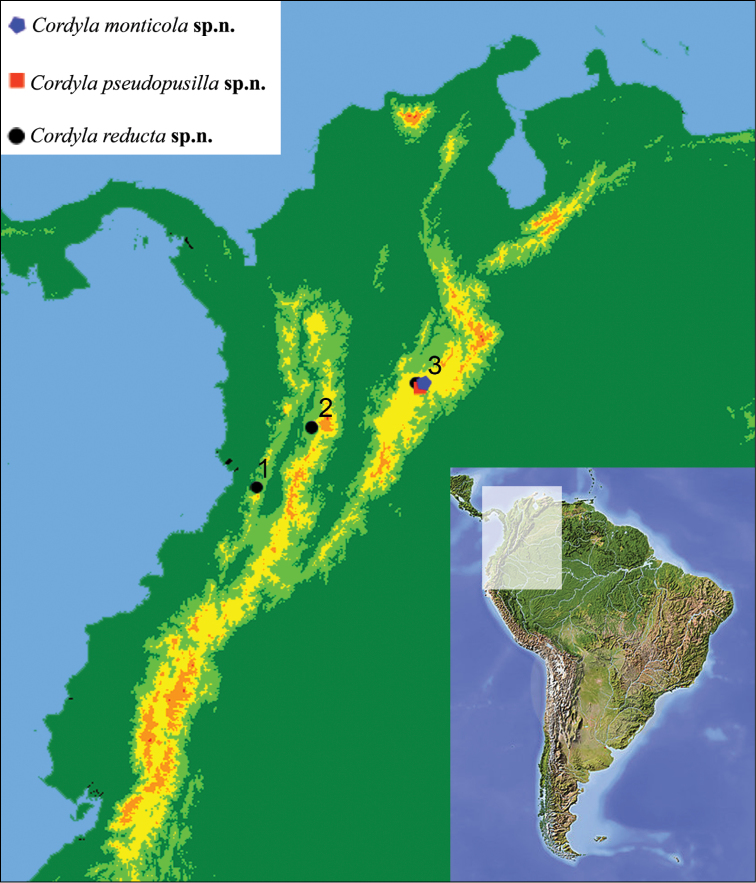
Collecting localities of *Cordyla* species in Colombian Andes. **1** “Parque Nacional Natural Farralones de Cali” **2** “Santuario de Flora y Fauna Otún Quimbaya” **3** “Santuario de Flora y Fauna Iguaque”.

Herein we present the occurrence of the genus *Cordyla* from the Colombian Andes (Fig. 38), representing the first described species in the Neotropical region. However, according to Vockeroth (2009: 276), unidentified specimens of *Cordyla* were previously known from Mexico, Belize, Guatemala, and Costa Rica. We were able to study the material housed at the Instituto Nacional de la Biodiversidad (INBio), San Jose, Costa Rica, that includes eight females from the provinces of Guanacaste, San José, Heredia, Puntarenas, and Limón (Vockeroth 2009; SSO pers. obs.). Because of the absence of associated male specimens, the formal description of species from that material has not been performed at the moment.

The presence of *Cordyla* in the north-western part of Colombia, in the Neotropical region, clearly corresponds to a secondary extension of a Nearctic clade to the south. The range of this extension is, however, restricted to the mountains of Colombian Andes. In spite of having studied a vast fungus gnat material from the Amazonian basin, the genus has not been recorded there (SSO, OK pers. obs.). This distribution pattern is actually not restricted only to *Cordyla* within the Mycetophilidae. Also *Docosia adusta* Oliveira & Amorim, 2011 belongs to a group of Nearctic mycetophilid species reaching Colombia. According to regionalization of the Neotropical region by Morrone (2014), two of the three localities (“PNN Farralones de Cali” and “SFF Otún Quimbaya”) lie in the Pacific Dominion and “SFF Iguaque” is located in the South American transition zone called Paramo province. *Cordyla reducta* sp. n. was recorded from all three localities while *Cordyla monticola* sp. n. and *Cordyla pseudopusilla* sp. n. are in the present stage of knowledge endemic to the Paramo province only (Fig. 38).

The complexity of the overlap of different biogeographical elements in Colombia was highlighted by Oliveira et al. (2007) and Oliveira and Amorim (2011). The Colombian fauna encompasses elements of Nearctic origin (p. ex. *Docosia adusta*), tropical origin, and a number of typical circum-antarctic elements that reach the north-western part of Neotropical region following the Andes final uplift to the north. The genera *Paraleia* Tonnoir, of which six new species were recently described from the Colombian Andes (Oliveira and Amorim 2012), and *Procycloneura* Edwards, of which at least ten species are being described by Oliveira and Amorim (in prep.) from Colombia and Costa Rica, are examples of amphinotic elements in Colombian fauna. Further studies of the diversity of Mycetophilidae and *Cordyla* in the Andes would contribute to a better understanding of the distributional patterns into the family and, hence, the biogeographical evolution of the region.

## Supplementary Material

XML Treatment for Cordyla monticola

XML Treatment for Cordyla pseudopusilla

XML Treatment for Cordyla reducta
